# O12.2. STICKS AND STONES MAY BREAK MY BONES BUT WORDS INCREASE THE RISK OF PSYCHOTIC EXPERIENCES

**DOI:** 10.1093/schbul/sby015.270

**Published:** 2018-04-01

**Authors:** Colm Healy, Mary Clarke, Ian Kelleher, Mary Cannon

**Affiliations:** Royal College of Surgeons in Irel

## Abstract

**Background:**

There has been a surge of interest into the relationship between psychotic experiences (PEs) and bullying. However, the methods of bullying and impact of bullying varies across individuals and the prevalence may also vary by respondent (parent or children). For this reason, a thorough investigation into this relationship is warranted.

**Methods:**

A longitudinal analysis was conducted on waves 1 and 2 (ages 9 and 13) of the nationally representative Growing Up in Ireland study. Data from n=7163 families were included in this study. Information regarding bullying, being a bully, bullying type, reasons for the bullying, the impact of the bullying was collected from the participating child and their primary care giver (PCG) at both waves. Psychotic experiences were reported by the child at the second wave using the Adolescent Psychotic Symptoms Screener.

**Results:**

13.12% of children met validated criteria for psychotic experiences. Based on the PCG’s account, 32.89% of those with PEs at age 13 were bullied at age 9 and this was independently associated with PEs even after accounting for bullying at 13 (OR: 1.40, CI: 1.19–1.65). Physical, verbal, electronic bullying and bullying by exclusion were associated with an increased risk of PE. However, in a multivariate analysis only verbal bullying was independently associated with an increased risk of psychotic experiences (OR: 1.56, CI: 1.27–1.93; adjusted for bullying at 13: OR: 1.47, CI: 1.19–1.82). There was a linear relationship between the number of different methods of bullying experienced at 9 and the risk of PEs at 13 (continuous OR: 1.24, CI: 1.14–1.34). Of the reasons for bullying given by the PCG, only ethnicity (OR: 2.36, CI: 1.46–3.80), being a teacher’s pet (OR: 2.09, CI: 1.17–3.73) and jealously (OR: 2.28, CI: 1.5–3.39) were significantly associated with PEs. Persistent bullying was associated with a higher risk of PEs relative to their peers (never bullied OR: 2.31, CI: 1.73–3.08; and bullied at one-time point: OR: 1.49, CI: 1.10–2.03).

Based on the child’s account, the vast majority of those who report being a bully (13.87%) at age 9 were also bullied (76.48%, OR: 7.04, 5.97–8.31). Both being a bully and being bullied at age 9 were associated with an increased risk of PEs (16.91%, OR: 1.34, CI: 1.09–1.64; and 50.48% OR: 1.71, CI: 1.48–1.98, respectively). In a multivariate analysis only being bullied was independently associated with PEs (OR: 1.68, CI: 1.44–1.96; adjusted for bullying at 13: OR: 1.57, CI: 1.34–1.83). Verbally bullying another was the only method of bullying associated with an increased risk of PEs at 13 (OR: 1.59, CI: 1.06–2.39). Of those reporting being bullied, verbal and written bullying at age 9 were associated with an increased risk of PEs at age 13 (OR: 1.25, CI: 0.97–1.6; and OR: 1.44, CI: 1.05–1.97, respectively). In a multivariate analysis only written bullying was associated with an increased risk of PEs (OR: 1.47, CI: 1.05–2.06; adjusted for bullying at 13: OR: 1.41, CI: 1.01–1.99). The impact of the bullying on well-being was also associated with an increased risk of PEs at 13 (OR: 1.36, CI: 1.09–1.72; adjusted for bullying at 13: OR: 1.30, CI: 1.04–1.63). Persistent bullying was associated with a vastly higher risk of PEs relative to their peers (never bullied: OR: 4.42, CI: 3.44–5.69; and bullied at one time point OR: 2.71, CI: 2.10–3.50).

**Discussion:**

Bullying is pervasive in the childhood of those who subsequent report PE. Bullying at age 9, particularly verbal and written bullying methods are risk factors for PEs in adolescence even when controlling for adolescent bullying. Persistent bullying was associated with a vastly higher risk of PEs. Reducing the rates of bullying in childhood may moderate the likelihood of PEs in adolescents.

